# Breastfeeding in preterm infants: public health policy in primary care

**DOI:** 10.1016/j.rppede.2016.03.011

**Published:** 2016

**Authors:** Joel Alves Lamounier

**Affiliations:** Department of Medicine, Universidade Federal de São João Del Rei (UFSJ), São Joao del Rei, MG, Brazil

The importance and benefits of human milk have been well established for full-term and also preterm children.^1^^,^ 2 However, breastfeeding rates are lower in preterm infants.^3^^,^ 4 The initial survival of premature infants depends on hospital care and adequate nutritional support. Human milk provides nutrients and protection elements against infection. After hospital discharge, the patient should be treated at specialized reference centers and breastfeeding should be maintained.^5^^-^ 7 It is important to identify mothers at risk of not breastfeeding and who delivered babies with very low and extremely low birth weight to schedule home visits and consultations at the basic health units (UBS), especially in the first weeks after hospital discharge.^8^^-^ 10 An observational study with preterm infants after discharge from the Neonatal Intensive Care Unit showed that support from the health teams at the UBS is required to maintain breastfeeding.^11^

In 2002, in the state of Minas Gerais, the Secondary Reference Viva Vida Centers (CVV) were established as a public policy aimed at reducing maternal and infant mortality. The CVV are public healthcare units, offering health care exclusively through the Brazilian Public Health Service (SUS), characterized as micro-regional average-complexity health care facilities, which must work in an integrated manner with primary and tertiary care, seeking to ensure full attention to sexual and reproductive health and provide care to at-risk children, including preterm ones. In the municipality of Viçosa, this was a successful, integrated program that included actions developed in a reference hospital for women with high-risk pregnancies, including the structure to provide care to preterm infants and nutritional support with a milk bank. After hospital discharge, the preterm infant has guaranteed care and follow-up at the CVV. The study by Freitas et al. shows the data from a retrospective cohort study of 103 preterm infants followed from 2010 to 2015 in this city. Breastfeeding rates were higher than that in full-term newborns in Brazil. In preterm infants younger than 37 weeks of gestation, the median duration of breastfeeding was 5.0 months, with a 2.6-fold higher risk of breastfeeding interruption in infants younger than 32 weeks. In preterm infants receiving supplemented human milk in the first visit after discharge, the risk of breastfeeding interruption was 3-fold higher, when compared with exclusive breastfeeding at the time. These good maternal breastfeeding indicators are the results of the integration of tertiary and primary care in the municipality.^12^

Breastfeeding promotion measures after hospital discharge, with adequate follow-up and comprehensive care to preterm infants reduce early weaning.^13^^-^ 15 Considering the increased survival of preterm infants, feeding difficulty is the main obstacle to overcome, what has been achieved with the improvement of care in neonatal units. However, it is still necessary to establish strategies to attain successful breastfeeding in primary care. This is a challenge that can be overcome with public policies aimed at the care of women from the prenatal and maternity periods to the outpatient treatment of preterm infants.
